# Effects of a long-term home-based exercise training programme using minimal equipment vs. usual care in COPD patients: a study protocol for two multicentre randomised controlled trials (HOMEX-1 and HOMEX-2 trials)

**DOI:** 10.1186/s12890-019-0817-7

**Published:** 2019-03-01

**Authors:** Anja Frei, Thomas Radtke, Kaba Dalla Lana, Julia Braun, Ramona M. Müller, Milo A. Puhan

**Affiliations:** Epidemiology, Biostatistics and Prevention Institute (EBPI), University of Zurich, Hirschengraben 84, 8001 Zurich, Switzerland

**Keywords:** COPD, Exercise training intervention, Randomised controlled trial, Home-based, Quality of life, Functional exercise capacity, Physical activity, Minimal equipment

## Abstract

**Background:**

Exercise training is an important component of pulmonary rehabilitation (PR) programmes in chronic obstructive pulmonary disease (COPD), but the great majority of COPD patients who would benefit from PR never follow such programmes or fail to maintain exercise training after PR completion. Against this background, we developed an exercise training programme that requires minimal equipment and can be implemented long-term in the patient’s home-setting. The aims of the HOMEX-1 and HOMEX-2 trials are to assess the effectiveness of this home-based exercise training programme in two groups of COPD patients over the course of one year: patients who have completed PR (HOMEX-1 trial) and patients who did not enrol in existing PR programmes within the last two years (HOMEX-2 trial).

**Methods:**

HOMEX-1 and HOMEX-2 are multicentre, parallel group, randomised controlled trials. For both trials each, it is planned to include 120 study participants with a diagnosis of COPD. Participants will be randomised with a 1:1 ratio into the intervention group or the control group (usual care/no intervention). The intervention consists of minimal-equipment exercise training elements with progressive level of intensity, conducted by the participant during six days per week and instructed and coached by a trained health care professional during three home visits and regular telephone calls during one year. Primary outcome is change in dyspnoea (domain of Chronic Respiratory Questionnaire) from baseline to 12-months follow-up. Secondary outcomes are change in dyspnoea over the course of the year (assessed at 3, 6 and 12 month) and change in functional exercise capacity, physical activity, health-related quality of life, health status, exacerbations and symptoms from baseline to 12 months follow-up. In addition, explanatory, safety and cost-effectiveness outcomes will be assessed. We will conduct intention-to-treat analyses separately per trial and per protocol analyses as sensitivity analyses.

**Discussion:**

The HOMEX-1 and HOMEX-2 trials assess a novel intervention that provides an innovative way of making exercise training as accessible as possible for COPD patients. If the intervention proves to be effective long-term, it will fill the gap of providing an easily accessible and feasible intervention so that more COPD patients can follow an exercise programme.

**Trial registration:**

ClinicalTrials.gov Identifier: HOMEX-1 NCT03461887 (registration date: March 12, 2018; retrospectively registered); HOMEX-2 NCT03654092 (registration date: August 31, 2018).

## Background

Exercise training is an important component of pulmonary rehabilitation (PR) and the management of COPD [1]. Numerous trials have shown that exercise training with or without additional PR elements leads to clinically significant improvements in health-related quality of life (HRQoL) and exercise capacity in patients with COPD [2]. However, despite the well-known benefits, the great majority of patients who would profit from PR never follow such a programme. On the other hand, for those patients who undergo a supervised exercise training programme, it is often challenging to maintain exercising after PR completion and to implement the training into the daily life home-setting. Major barriers for not participating or adhering to programmes are travel and transport to the centre, disruption to established routine, lack of perceived benefits, social isolation, inconvenient timing, comorbidities and depressive symptoms [3–7].

Traditionally, PR programmes were given under direct supervision at a rehabilitation centre with participants attending as inpatients or outpatients. However, both community-based and home-based programmes are becoming increasingly popular [1]. Of the studies included in the most recent systematic review on the effects of PR vs. usual care in COPD patients [2], approximately one third comprised home-based programmes or programmes that included an element of home-based training embedded in an inpatient or outpatient setting. Of the studies that focused on home-based training alone, the programmes typically lasted eight to twelve weeks, the follow-up assessments were primarily conducted at the end of the programme without a more long-term perspective and their effect on HRQoL and exercise tolerance compared to usual care was not consistent across studies [8–17].

Evidence from two recent trials suggests that the effects of comprehensive home-based PR programmes were equivalent or non-inferior to the effects of outpatient PR, both after completion of the programmes (12 and 8 weeks, respectively) and after one year [18, 19]. These results were supported by a current systematic review that found similar beneficial effects of home-based compared to outpatient exercise training programmes [20]. However, as demonstrated in other studies [1, 21], in both, the home-based and outpatient PR groups the strong effects detected immediately after completion of PR either decreased [18] or vanished in the long-term after 12 months [19]. Another recent trial showed beneficial effects of a long-term maintenance exercise training programme (following PR) after two years; however, the programme included use of cycle ergometers which were delivered to the participants’ home, and weight training. Moreover, patients visited the hospital for supervised exercise training sessions on a regular basis [22].

Against this background, we developed a novel exercise training programme that requires only minimal equipment and that can be easily implemented in the patient’s home-setting in the long-term (HOMEX intervention). The overall objectives of the HOMEX-1 and HOMEX-2 trials are to assess the effectiveness of this intervention in two groups of COPD patients by means of two randomised controlled trials (RCT). The HOMEX-1 trial focuses on patients who completed PR no longer than one month ago, aiming at maintaining exercise training effects elicited during PR for the long-term (post PR maintenance group). The HOMEX-2 trial focuses on patients who did not participate in PR within the last two years aiming to offer a low-threshold complementary option for regular exercise training in their home setting (no PR group). The primary objective of the studies is to assess the effect of the HOMEX intervention on dyspnoea over the course of one year; secondary objectives are to evaluate the effect of the intervention on functional exercise capacity, physical activity, health status, exacerbations and COPD associated symptoms. Additional objectives are to explore the patients’ compliance and adherence to the exercise training programme and to evaluate acceptability, implementation feasibility and cost-effectiveness of the intervention.

## Methods

### Study design

HOMEX-1 and HOMEX-2 are multicentre, randomised (ratio 1:1), parallel group, controlled trials (Clinicaltrials.gov Identifier: NCT03461887 and NCT03654092). Study participants are randomly assigned either to the home-based exercise group (intervention group) or to no intervention/usual care (control group). Both trials were approved by the local ethics committees (Kantonale Ethikkommission Zürich, Ethikkommission Bern, Ethikkommission Zentral- und Nordwestschweiz; BASEC-Nr. 2017–02092) and are conducted in accordance with the Declaration of Helsinki and the EC/ICH-Guidelines on Good Clinical Practice. The study is restricted to participants who provided written informed consent.

### Study setting, study population and recruitment

For HOMEX-1, eligible participants are identified from the Swiss inpatient PR centres Klinik Barmelweid, Berner Reha Zentrum Heiligenschwendi and Zürcher RehaZentrum Wald, and the outpatient PR centre Kantonsspital Winterthur. All patients admitted to the PR centres will be screened for study eligibility by the local investigators. Eligible patients will be consecutively informed about the study and invited to participate. After written informed consent, baseline assessments will be conducted. Recruitment for HOMEX-1 is planned for 12 months; the first participant in the first centre was enrolled by the end of January 2018.

For HOMEX-2, study participants will be primarily recruited in the Canton of Zurich. Potential participants will be informed about the study by means of information flyers including a phone number of a contact person of the Epidemiology, Biostatistics & Prevention Institute (EBPI), University of Zurich, through direct contact with LUNGE ZURICH staff (local Lung Association of the Canton of Zurich), general practitioners and other health professionals. Additional information on the study is provided in newsletters of LUNGE ZURICH and/or advertisements and the EBPI homepage. EBPI study staff will consecutively conduct screening visits at the EBPI in Zurich, where potential participants will be screened for eligibility and receive oral and written information. A pulmonologist will confirm COPD diagnosis based on a lung function measurement (portable hand-held spirometry) and assessment of lifetime smoking and occupational exposure. In case of any uncertainties regarding COPD diagnosis, the study team will contact the participants’ general practitioner or pulmonologist. After written informed consent, baseline assessments will be conducted. Recruitment for HOMEX-2 will start by the end of September 2018 and is planned for 12 months.

#### Inclusion criteria

##### HOMEX-1 and HOMEX-2

- Signed informed consent
- Diagnosis of COPD, defined as forced expiratory volume in 1 s/forced vital capacity (FEV1/FVC) ratio < 0.7 and FEV1 < 80% predicted after bronchodilation, with or without chronic symptoms (cough, sputum production) corresponding to GOLD stages II-IV
- Male and female participants ≥40 years of age
- Knowledge of German language to understand study material and assessments

##### HOMEX-1 only

- Completion of an inpatient or outpatient PR no longer than 1 months ago

##### HOMEX-2 only

- No participation in an inpatient or outpatient PR within the last two years

#### Exclusion criteria

##### HOMEX-1 and HOMEX-2

- Participants not able to conduct the exercise training programme due to physical, cognitive or safety reasons, as judged by investigator; e.g., lower limb joint surgery within preceding three months, unstable cardiac disease, predominant neurological limitations

##### HOMEX-2 only

- Planned participation in a PR programme

### Study groups

Participants assigned to the intervention group will receive the HOMEX intervention (minimal equipment exercise programme; described below), participants assigned to the control group will not receive the intervention (usual care). Apart from receiving the HOMEX intervention or not, the study does not impact any other planned treatments.

### Study intervention

The exercise training programme HOMEX was developed by health professionals and experts from the EBPI and thoroughly piloted in several COPD patients by means of cognitive debriefing techniques (study material) and by evaluation of the motivational counselling strategy, instruction, conduct and feedback of the exercises and optimal scheduling of the home visits and follow-up calls.

To consider the challenge of conducting regular exercise training in the long-term, we developed the intervention under consideration of important determinants of health behaviour. We therefore aimed to instruct and coach the minimal equipment exercise training programme to participants based on motivational interviewing techniques and with support of self-management skills like problem solving, decision making and action planning. The comprehensive coaching targets the participant’s behaviour at various levels; i.e., the individual level (e.g., personal goal setting), interpersonal level (e.g., social support) and environmental level (e.g., own flat / house) [23].

The study intervention consists of three visits at the participant’s home by a trained health care professional, the “personal coach” (mainly physiotherapists, but also other professions; training and supervision described below), exercise sessions conducted during six days per week, illustrated by cards, an interactive exercise training book and supporting telephone calls. Additional intervention elements are that a relative, friend or close person is involved as a “sparring” partner to support the participant to conduct the exercise training programme on a regular basis and that the general practitioner is informed about the participation of his/her patient in the intervention.

#### Initial visit at the participant’s home

At programme onset, the personal coach conducts an initial visit at the patient’s home (approx. 120 min). The aims of this visit are to establish trust and confidence, to explore the participant’s ability to perform exercises and to instruct him/her for the first part of the exercises and the use of the exercise cards. Besides the cards, the coach introduces the participant also to the daily use of the interactive training book in order to report individualised goals/rewards, record and evaluate daily workouts and define concrete motivational resources. Furthermore, the concrete training setting at home, i.e., the precise location of the chair and the training schedule will be determined. The communication based on motivational interview techniques aims to ensure a high level of training adherence throughout the study. The patient is also instructed to call his coach upon interruptions of training for more than three consecutive days due to any reason.

#### Second home visit after three weeks

The aim of the second visit is to further build on trust and confidence, to instruct the participant in the remaining exercise cards, to verify exercise performance, to ensure the regular adaption in training volume and training intensity, to check the daily entries in the training book and to discuss and adapt with the participant short and long term goal attainment.

#### Third home visit after eight to nine weeks

The aim of the third home visit is to ensure open and honest communication, the appropriate performance of all exercises, to check and ensure the regular adaption in training volume and training intensity, to discuss and monitor the first short term goal achievement and to discuss and possibly adapt the long-term goal.

#### Individualised strength-training programme

The exercise training consists of three trunk, three upper limb and four lower limb exercises that can be performed at three different levels. The exercises will be performed six days per week for about 15–20 min using a chair and elastic exercise bands on different resistance levels. The daily strength training is always introduced by a whole body warm-up and finalised by two or three appropriate stretching exercises. During the programme, the intensity of exercises will progressively increase. All exercises are illustrated on 38 attractive exercise cards including possible gain, detailed instructions to verify the correct performance, training volume and intensity. To document the training progress, the participants will record their completed training sessions in their training book which will also be used to monitor training adherence.

#### Regular telephone calls

During the 12-month exercise training programme the personal coaches will regularly call the participants, directed by a guidance manual to motivate the participants, to discuss training progress and concrete benefits and barriers and to adapt goals and the training programme (exercise intensity, duration) to the individual needs, if required. During the first ten days, the coach will call the participant twice, subsequently once every two weeks for the first 6 months and every five weeks after, except for situations when training elements have changed. Participants are instructed to call their coach if they could not perform the exercises for more than three consecutive days (i.e., except Sundays), they will then be followed by additional phone calls. To ensure and guide the training restart, additional phone calls will be conducted, with similar frequency as after the first home visit. Since motivational components are a key issue for programme success, the telephone calls will be based on motivational interview techniques and target on support in self-management skills to facilitate a high level of training adherence.

#### Training and supervision of health care professionals (“personal coach”)

The health care professionals who will work as personal coach will be trained by the master coach (KDL, who was much involved in developing and piloting of the intervention) during a basis and an update training session. For each participant contact (i.e., all 3 home visits and the telephone contacts), the coach will be provided with guidance documents with algorithms how to proceed in specific situations. During the time of the intervention, the coach will be in regular contact with the study team as well as part of a virtual chat room with all other coaches moderated by members of the study team and supported by the master coach. The master coach will accompany the coach at least once at a home visit to ensure the conduct of the intervention according to protocol (fidelity).

### Study outcomes

Main study outcomes are assessed during study visits before randomisation at baseline (T0, T1) and at 12-months follow-up (T4). Further assessments are conducted after 3 months (T2) and after 6 months (T3). Table 1 displays the study visits and assessment of the outcomes.

**Table 1:**
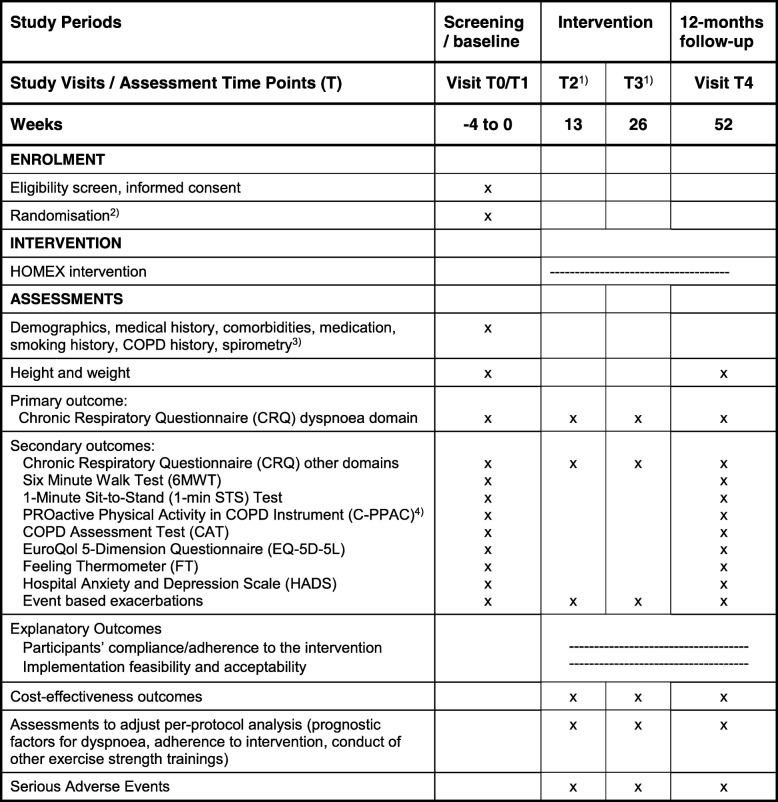
Schedule of study visits and assessments

1) For T2 and T3 assessments, a questionnaire will be sent by mail to participants. At T3, study staff will also call the participants to verify in detail the questions regarding cost-effectiveness and other exercise training

2) Randomisation will be conducted at the end of visit T1, after all assessments have been conducted

3) HOMEX-1 participants: measurements are already collected during rehabilitation; HOMEX-2 participants: a lung function measurement will be conducted by a handheld spirometer

4) HOMEX-2 only

#### Primary outcome

The primary outcome is the change in dyspnoea, measured by the Chronic Respiratory Questionnaire (CRQ) dyspnoea domain (standardised version, self-administered) from baseline until 12-months follow-up [24]. The CRQ dyspnoea domain is one of the most commonly used dyspnoea instrument in trials of PR and contains five questions responded on a 7-point Likert-type scale ranging from 1 (most severe dyspnoea) to 7 (no dyspnoea).

#### Secondary outcomes

Secondary outcomes are assessed at baseline and at 12-months follow-up, except for change in CRQ dyspnoea subscale, which is additionally assessed at 3 and 6 months follow-up.

#### Functional exercise capacity

The *Six-Minute Walk Test (6MWT)* [25, 26] is a validated and widely used test in COPD patients to assess functional exercise capacity. The test will be performed according to European Respiratory Society (ERS)/American Thoracic Society (ATS) standards [25, 27]. The patients will be instructed to walk as far as possible during 6 min and they will receive standardized encouragements. Two tests will be performed with at least 30 min rest in-between and the test with the higher total walking distance in meters will be recorded.

The *1-Minute Sit-to-Stand Test (1-min STS test)* is a validated test to assess functional exercise capacity in individuals with COPD [28]. It counts the number of repetitions of full sit-to-stand movements from a standard chair (i.e. standing up and sitting down again) that a person completes during one minute. The STS movement requires the participant to stand up completely vertical until in the straight upright position and return to the sitting position with knees flexed to approximately 90 degrees. Prior to the STS test participants will practice the STS movement once or twice in order to familiarise themselves.

#### Health-related quality of life (HRQoL)

HRQoL will be assessed by the *CRQ* domains *fatigue, emotional function and mastery* [24]. The whole CRQ (including the dyspnoea domain) contains 20 questions responded to on a 7-point Likert-type scale, ranging from 1 to 7 with lower scores indicating worse HRQoL. HRQoL will additionally be measured by the *EuroQol 5-Dimension Questionnaire (EQ-5D-5 L)* (EuroQoL Group, www.euroqol.org) including a descriptive system for five dimensions (mobility, self-care, usual activities, pain/ discomfort, anxiety/depression) as well as a visual analogue scale for overall health state (feeling thermometer, see below) [29].

#### Physical activity

Physical activity will be assessed by the *clinical visit version of PROactive Physical Activity in COPD Instrument (C-PPAC)* in HOMEX-2 participants only. The C-PPAC is a validated and reliable hybrid tool combining a short patient-reported outcome questionnaire and two activity monitor variables (assessed by the ActiGraph® accelerometer, Pensacola, FL, USA) to measure physical activity in COPD patients in the two domains amount and difficulty (item scores 0–4, scale 0–100) [30, 31]. We will measure physical activity during one week prior to the baseline and the 1-years follow-up visit.

#### Health status and symptoms

Health status and symptoms will be assessed by the *COPD Assessment Test (CAT)* which measures the impact of COPD on a person’s health status (8 questions, 6-point Likert-type scale) [32], the *feeling thermometer (FT)*, a visual analogue scale for overall health state ranging from 0 (worst health you can imagine) to 100 (best health you can imagine) [33, 34], and of the *Hospital Anxiety and Depression Scale (HADS)* which assesses symptoms of depression and anxiety (14 questions, 4-point Likert-type scale) [35].

#### Event based exacerbations

Exacerbations are assessed patient-reported at 3-, 6- and 12-months follow-up. Event-based definition requires an increase in symptoms and an increase in dosage of or new prescription of systemic corticosteroids and/or antibiotics.

#### Explanatory outcomes

*Participants’ compliance and adherence to the exercise training programme* (intervention group participants only) is assessed patient-reported as the percentage of fulfilled training sessions according to the participants’ handwritten reports in their exercise training books. To minimise information bias, participants will be informed by their coaches to fill-in the training books regularly and truthfully. Furthermore, compliance is assessed from the health care professionals’ perspective by evaluating coaches’ reports regarding exercise training interruption of more than three consecutive days.

*Implementation feasibility and acceptability* including satisfaction and experience with the exercise training programme and health professionals’ feedback on the intervention will be assessed by a satisfaction questionnaire and a brief semi-structured interview (participants) and/or focus groups (with participants and health professionals separately) at 12-months follow-up. In addition, fidelity to programme delivery will be assessed during the intervention period by the master coach.

#### Safety outcomes

*Serious adverse events (SAEs)* that occur after baseline are sought by non-directive questioning of all participants at 12-months follow-up. SAEs are also detected when they are volunteered by the participants during the course of the study and, for intervention group participants, at every contact. They are documented, their causal relationship with the intervention is assessed and they are reported to the responsible ethics committee according to the legal requirements.

#### Assessments to adjust per-protocol analysis

The per-protocol analyses will be adjusted based on a new approach proposed by Miguel Hernán [36] with the following a priori specified factors: 1) *Prognostic factors for the primary outcome dyspnoea*, 2) *Adherence to conduct the exercises*, and 3) *Information whether the participants conducted other strengthening exercise trainings* (for details see chapter Statistical analyses). Besides the prognostic factors which are already assessed by routinely planned measurements, we will additionally assess self-efficacy to conduct the exercise training and information on other exercise strength trainings by the participants.

#### Cost-effectiveness outcomes

We will conduct health economic analysis comparing the relative costs and outcomes (effects) of the intervention and the comparator. Outcomes are assessed after 3, 6 and 12-months and include *1) Direct healthcare costs* (patient-reported: physician visits, specialist visits, number of hospitalisations, total number of hospitalisation days, number of days in rehabilitation / study-staff-reported: costs of working hours (administration of the intervention, trainings, supervision of the intervention), costs of intervention material (elastic bands, training book); *2) Direct non-healthcare costs* (patient-reported: ambulance transportations to hospital (travel costs to usual physician visits are not considered), nursing home care); and 3) *Indirect costs; mainly productivity loss costs* (patient-reported: working status and disability status due to COPD, workdays lost due to COPD and related adverse events).

### Study visits and procedures

**Fig. 1:**
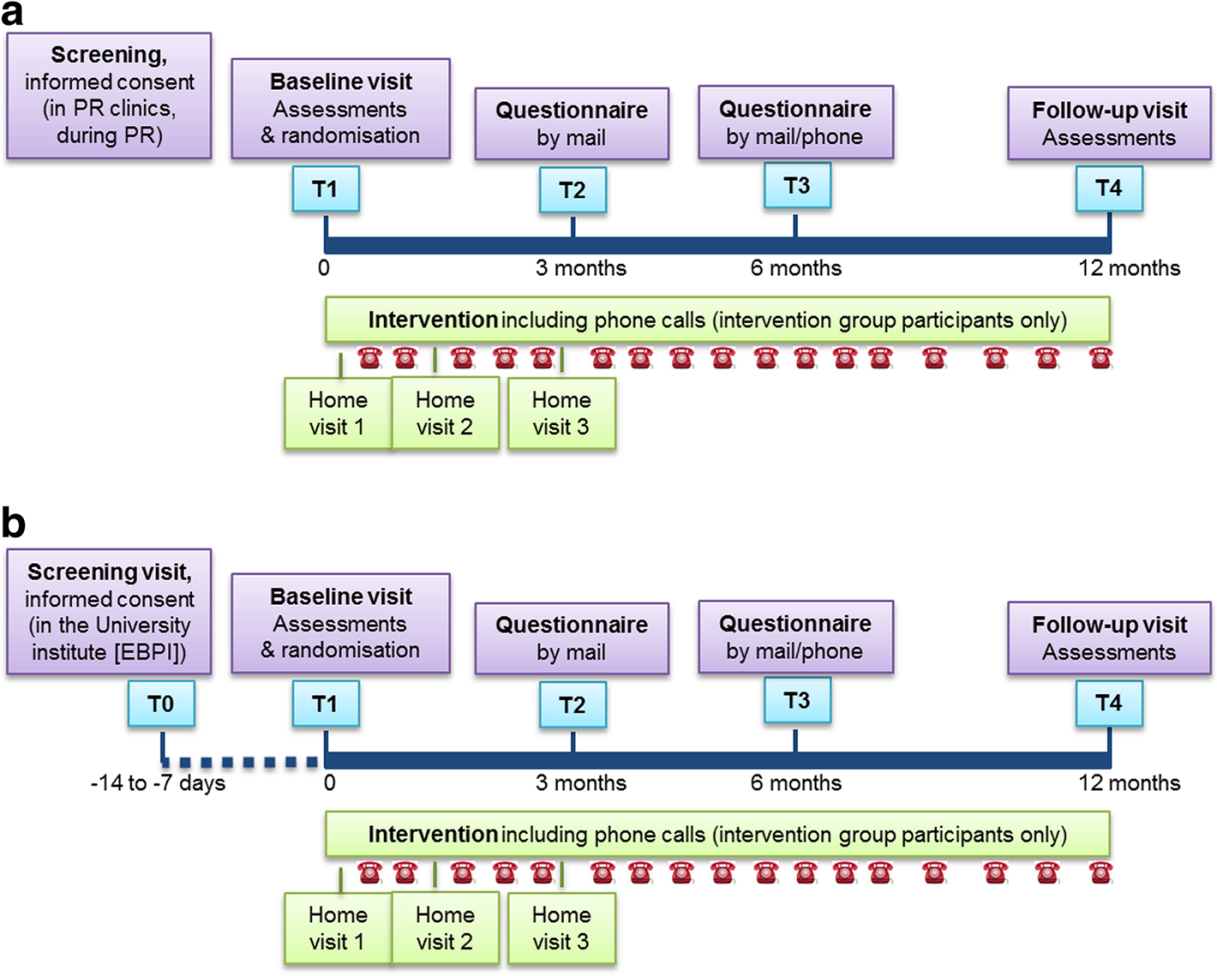
Study flow per participant and overview on time schedule of study visits, assessments and intervention (1a: HOMEX-1, 1b: HOMEX-2)

In the course of the study, assessment visits are planned at baseline (one visit for HOMEX-1 [T1] and two visits for HOMEX-2 [T0 and T1]) and at 12-months follow-up (T4). The visits are conducted by trained study nurses at the same location; for HOMEX-1 at the PR clinics where the study participants were recruited, for HOMEX-2 at the EBPI in Zurich. In addition, after 3 (T2) and 6 (T3) months a questionnaire including the CRQ, cost-effectiveness outcomes, assessment of other exercise trainings and prognostic factors for dyspnoea will be sent by mail to all participants. At 6 months follow-up, study staff will additionally call the participants to verify in depth responses regarding cost-effectiveness and other exercise training. Figure 1 (1a: HOMEX-1, 1b: HOMEX-2) shows the study flow per participant and the time schedule of study visits, assessments and intervention.

### Randomisation

Participants will be randomised for each HOMEX-trial separately, on the level of participants, with a 1:1 ratio, using block randomisation with varying block sizes, stratified by numbers of repetitions in the 1-min STS test (≤19 vs. > 19 repetitions) and, for HOMEX-1 only, additionally stratified by study centre. Randomisation is performed by means of Research Electronic Data Capture (REDCap) [37], a secure, web-based application specifically designed to support data capture for research studies which ensures concealment of random allocation. A biostatistician from the EBPI who is not involved in the study created for both HOMEX-trials a separate randomisation list using nQuery Advisor® + nTerim® 4.0 (Statsols, Cork, Ireland). Another independent researcher from the EBPI implemented these lists into the REDCap database. Randomisation will only take place after all baseline assessments are completed.

### Blinding procedures

Due to the kind of intervention, neither participants nor health care professionals can be blinded after the participants’ assignment to the intervention or control group. Data analysts will be blinded to group assignment.

### Withdrawal/discontinuation of participants

If a participant withdraws consent, the primary reason for this decision will be recorded. The exercise training programme will be discontinued and no further assessments conducted. If a participant is unable to continue the intervention for an extended period of time (assessed by the investigators regarding the specific participant and bearing in mind the participant’s personal situation), the participant is not considered withdrawn from the study. If he/she agrees, 12-months follow-up assessments will be conducted; if he/she refuses, follow-up assessments will be conducted at the time of discontinuation. Compliance/adherence to the study intervention will be analysed by evaluation the training books, the conduct of the home visits and regular phone calls and the records of the coaches’ reports.

### Data management

Data will be collected and managed using REDCap database hosted at the Clinical Trials Center (CTC) at the University Hospital Zurich, Switzerland. Study nurses, coaches and designated investigator site staff will enter the data; they will not be given access to the system until they have been trained. Automatic validation procedures within the system check for data discrepancies during and after data entry and, by generating appropriate error messages, allow the data to be confirmed or corrected online by the designated investigator site staff.

### Monitoring and quality assurance

All study nurses are trained before study onset, a second training will take place prior to the first 12-months follow-up visit. EBPI study staff will conduct three monitoring visits at each study centre; one 4 to 8 weeks after the conduction of the first baseline visit, a second after completion of all baseline assessments and a third after completion of all 12-months follow-up assessments. During these visits, the monitor will participate in one visit and verify whether the participants’ instruction, data assessment and randomisation are conducted according to protocol and check whether study procedures, documentation and REDCap data entry are conducted correctly. The monitor will check data entries of the primary and secondary outcomes in the REDCap database. The monitoring records are discussed with the Principal Investigator and, if necessary, procedures are derived to improve the quality of data collection, processes, documentation and data entry.

All coaches are fully trained before intervention onset. During the intervention a regular exchange between coaches takes place by a protected forum. In addition, every 3 to 6 months meetings with all coaches are conducted for exchange purposes. The master coach accompanies all coaches during one of their first home visits, checks whether the elements of home visits have been carried out according to the protocol and gives feedback to the coaches.

### Sample size

For each of the two trials, the sample size calculation is based on the primary outcome change in the CRQ dyspnoea domain from baseline to 12-months follow-up. For HOMEX-1, the hypothesis is maintenance in intervention group and decline in control group; for HOMEX-2, the hypothesis is improvement in the intervention group and maintenance in control group. The well-established minimal important difference of the CRQ dyspnoea scale is 0.5 [38]. Assuming a standard deviation of the outcome variable of 0.9 [2] (according to the literature we assumed the same standard deviation of CRQ dyspnoea outcomes for both patient populations, for those recently completing PR and for those who did not complete PR within the last 2 years), 80% power and a significance level of 5% (two-sided), a sample size of 52 patients in each group is required which results in a total sample size of 120 per trial, including a drop-out rate of 15% (patients for whom follow-up assessments are not possible; sample size calculator: http://hedwig.mgh.harvard.edu/sample_size/js/js_parallel_quant.html).

### Statistical analyses

Analyses are conducted per trial separately. Baseline characteristics of the study participants are summarised according to numbers and percentages for qualitative variables, mean and standard deviation for quantitative variables with normal distribution and median and 25th–75th percentiles for quantitative variables with non-normal distribution. Differences in the change of the primary outcome CRQ dyspnoea from baseline to 12-months follow-up between the intervention and control group participants are compared by linear regression analyses corrected for stratification variables and, if deemed necessary due to baseline imbalances, potential confounders. The same analyses are used for continuous secondary outcomes. Changes in CRQ dyspnoea over the course of the year (assessed at 3, 6 and 12 month) will be examined using linear mixed models. The exploratory outcomes compliance to the training, satisfaction and health professionals’ feedback as well as the implementation process parameters will be analysed with a mixed-methods approach (using quantitative and the appropriate qualitative methods). If there is a substantial amount of missing values, we will compare the results from the complete case analysis with the results from a multiple imputation approach.

For the main analyses, we will use an intention-to-treat approach, i.e., the study participants will be analysed according to the group which they were originally randomised to, regardless of whether they adhered to the intervention according to protocol. Missing follow-up data of participants who did not conduct 12-months follow-up assessments will be substituted by the last available measurement (last observation carried forward).

As a sensitivity analysis, we will additionally conduct a per-protocol analysis, where we will keep only those participants in the intervention group who adhered to the intervention in terms of conducted exercise trainings [36]. We define adherence to protocol as “conduct of exercise training sessions at least during 70% of the weeks within the year of intervention the participants were able to train at least during 4 times a week”, i.e., weeks when participants suffered from pulmonary exacerbations or other serious health conditions that prevented them from training are not considered; as discussed with the coach (definition of a fulfilled individualised training session per day [according to protocol: 3 different exercises per day are planned]: conduct of at least 2 different exercises per day / definition of a fulfilled week of training [rate of training sessions according to protocol: 6 per week]: conduct of at least 3 out of the planned 6 individualised training sessions per week on average).

We will adjust the per-protocol analyses with prognostic factors for the primary outcome dyspnoea (dyspnoea, lung function [FEV_1_], exercise capacity and events of exacerbations) and for adherence to conduct the exercises (self-efficacy to conduct the exercises, exercise capacity, dyspnoea and acute worsening of health state [exacerbations and other]) and with the information whether the patients conducted other exercise strength trainings. Concerning the control group, we will collect as much information as possible, especially with respect to the type and frequency of self-organised exercise training. This will also be included in the per-protocol analysis.

The cost-effectiveness of the intervention and comparator will be assessed as cost per quality-adjusted life year (QALY) gained. The analysis will be performed using a time horizon of one year (i.e., equal to the maximal follow-up of the trial) and using a societal perspective. No interim analyses are planned.

## Discussion

The HOMEX-trials addresses two important and long-existing gaps in the context of PR. First, the study intervention provides an innovative way of making exercise training as accessible as possible for COPD patients by introducing a minimal equipment and home-based exercise training programme together with a number of components that address important determinants at the individual level, interpersonal level and environmental level for performing regular exercise training. Second, in contrast to many exercise training programmes that have been tested so far, the study intervention has a long-term perspective. It is important to highlight that the study intervention aims to achieve long-term adherence to exercise training by providing a home-based programme that is supported by health care professionals and others close to the patients in order to address important determinants for performing exercise training.

If our home-based minimal equipment exercise training programme proves to be effective long-term, it will fill the important gap of providing an easily accessible and feasible option so that more COPD patients can follow an exercise programme than it is currently the case. Furthermore, it will also provide much needed evidence on how exercise training can be maintained in the long-term. Thus, the HOMEX trials will address two key scientific questions in the context of PR for patients with COPD.

In Switzerland, the Cantonal Lung Associations that are covering the county already provide services both at their centres and the patients’ homes. The proposed intervention has great potential to supplement existing services with a long-term home-based exercise training programme option for COPD patients who either are not willing or not able to participate in existing centre-based programmes. The Swiss Lung Association could support the implementation by organizing and providing training and certification of health care professionals as well by supporting communication and dissemination in their patient population. Fortunately, the Swiss Lung Association has already expressed interest in taking over our home-based minimal equipment exercise training programme and supports its implementation, because it fits well with their strategic goal of providing important patient-centred services for individuals with COPD.

## Abbreviations

1-min STS: 1- Minute Sit-to-Stand Test
6MWT: Six- Minute Walk Test
ATS: American Thoracic Society
CAT: COPD Assessment Test
COPD: Chronic Obstructive Pulmonary Disease
CRQ: Chronic Respiratory Questionnaire
EBPI: Epidemiology, Biostatistics and Prevention Institute, University of Zurich
EQ-5D-5L: EuroQol 5-Dimension Questionnaire
FEV_1_: Forced expiratory volume in 1 s
FVC: Forced vital capacity
HADS: Hospital Anxiety and Depression Scale
HRQoL: Health-related Quality of Life
PR: Pulmonary Rehabilitation
QALY: Quality adjusted life year
SD: Standard Deviation
